# Screening and Interaction Analysis Identify Genes Related to Anther Dehiscence in *Solanum melongena* L.

**DOI:** 10.3389/fpls.2021.648193

**Published:** 2021-07-22

**Authors:** Zhimin Wang, Chao Yuan, Shaowei Zhang, Shibing Tian, Qinglin Tang, Dayong Wei, Yi Niu

**Affiliations:** ^1^College of Horticulture and Landscape Architecture, Southwest University, Chongqing, China; ^2^Key Laboratory of Horticulture Science for Southern Mountains Regions, Ministry of Education, Chongqing, China; ^3^The Institute of Vegetables and Flowers, Chongqing Academy of Agricultural Sciences, Chongqing, China

**Keywords:** eggplant (*Solanum melongena* L.), transcriptome, phytohormone, gene expression, interaction

## Abstract

Anther indehiscence is an important form of functional male sterility that can facilitate the production of hybrid seeds. However, the molecular mechanisms of anther indehiscence-based male sterility in eggplant (*Solanum melongena* L.) have not been thoroughly explored. We performed transcriptome sequencing and real-time quantitative reverse transcription-PCR (qRT-PCR) assays to compare the fertile line (F142) and male sterile line (S12) eggplant. We identified 2,670 differentially expressed genes (DEGs) between lines. Gene ontology (GO) and Kyoto Encyclopedia of Genes and Genomes (KEGG) pathway analyses identified 31 DEGs related to hormone biosynthesis. We, therefore, measured phytohormone contents, such as jasmonic acid (JA), auxin (IAA), gibberellin (GA), and abscisic acid (ABA) in S12 and F142. There were differences in IAA, GA_3_, and ABA levels between S12 and F142, while JA levels were significantly lower in S12 than in F142. Five key genes in the JA signaling pathway were differentially expressed in S12 vs. F142. Of these, *SmJAZ1* and *SmJAR1* were significantly upregulated and *SmDAD1, SmLOX*, and *SmCOI1* were downregulated in S12 vs. F142. Protein–protein interaction studies identified a direct interaction between SmDAD1 and SmLOX, while SmDAD1 failed to interact with SmJAR1, SmCOI1, and SmJAZ1. The data represent a valuable resource for further exploration of regulatory mechanisms underlying anther dehiscence in eggplant.

## Introduction

Eggplant (*Solanum melongena* L.), a popular vegetable crop that is thought to have originated in Africa, is widely cultivated in Africa, Asia, Europe, and the Near East (Bohs, 2010). The major characteristic of eggplant exhibits obvious heterosis, and the early use of hybrid vigor in the breeding of eggplant cultivars has been described (Kakizaki, 1931; Rodríguez et al., 2008). The use of reliable male-sterile systems could simplify the process and reduce the labor, cost, and time involved in producing hybrid eggplant seeds (Mennella et al., 2010). Functional genic male sterility (GMS) was reported in eggplant in 1954 and 1963 (Jasmin, 1954; Nuttall, 1963). The first functional male sterile eggplant mutant UGA 1-MS (Phatak and Jaworski, 1989) was discovered in 1989 and further characterized in 1991 (Phatak et al., 1991), and this kind of sterility was also further explored in 2009 as the result of crossing eggplant with wild relatives (Khan and Isshiki, 2009, 2010). The GMS line showed anther indehiscence in which the anthers did not open to release pollen, thereby disabling pollination (Wang et al., 2021). Anther dehiscence is a vital process in which mature pollen grains are released from the locules of the anther, thus enabling pollination (Sanders et al., 1999, 2005). Although morphological changes in anthers during dehiscence have been thoroughly described (Beals, 1997; Sanders et al., 2005), the molecular mechanisms controlling anther dehiscence remain relatively unknown.

Jasmonic acid (JA) is a lipid-derived hormone that functions as an important regulator of plant responses to various stresses as well as development (Scott et al., 2004). Analyses show that JA affects wheat development, including germination, growth, flowering time, senescence, and alters tolerance to environmental stresses (Wasternack and Strnad, 2018). Wheat plants with high JA levels are characterized by delayed germination, slower growth, late flowering, and senescence, and improves tolerance to short-term freezing (Nausica et al., 2007). The application of exogenous jasmonate significantly stimulates root hair elongation (Bohs, 2010). JA also plays an important role in regulating anther dehiscence (Ishiguro et al., 2001; Xiao et al., 2014).

Jasmonic acid signal transduction pathways have been investigated in *Arabidopsis thaliana* (Schaller and Stintzi, 2009; Wasternack and Strnad, 2018). JA biosynthesis originates from fatty acids in chloroplasts, and then its metabolic compounds are produced from 12 different pathways in peroxisomes and cytosol, respectively (Wasternack and Strnad, 2018). Gene mutations involved in JA biosynthesis cause failure or delay of anther dehiscence and may lead to male sterility. Several of these genes have been identified, such as anther dehiscence defect 1 (*DAD1*) (Ishiguro et al., 2001), *AOS* (Bae et al., 2010), *LOX* (Caldelari et al., 2011), *COI1* (Xie et al., 1998), *DEHISCENCE 1* (*DDE1*), /*OPR3* (Schaller et al., 2000; Stintzi and Browse, 2000), and the triple mutation (*fad3, fad7*, and *fad8*) (Mcconn and Browse, 1996). In addition, *JAR1* (a JA-amino acid synthetase) has a biological function in regulating flower opening and closure, and anther dehiscence in rice (Xiao et al., 2014). Some studies have found possible mechanisms that JA was a significant regulator of anther dehiscence. The JA pathway genes *SmJAZ1* and *SmOPR3* are downregulated in the male sterility S16 (Zhang et al., 2020). Moreover, SmOPR3 could interact with the transcript accumulation of the eggplant CORONATINE INSENSITIVE1 (SmCOI1) to form a protein complex, and COI1 interacts with JAZ1 in the presence of JA-Ile (Zhang et al., 2020). Through activating *DAD1* in *Arabidopsis*, a RING-type E3 ligase controls anther dehiscence (Peng et al., 2013). However, the exact mechanisms of JA activity regulating anther dehiscence in eggplant remain to be elucidated.

In this study, we performed transcriptome analysis to identify differentially expressed genes (DEGs) in eggplant S12 (indehiscent anthers) and F142 (dehiscent anthers) in order to uncover differences in the anther dehiscence network. Enrichment analysis of the DEGs and endogenous hormone measurements highlighted the effect of JA signal transduction pathways in anther dehiscence. Finally, we analyzed the relationships between five genes in the JA pathway by yeast two-hybrid (Y2H) analyses. The results lay the foundation for further uncovering the molecular mechanisms and biological function of anther dehiscence in eggplant.

## Materials and Methods

### Plant Materials and Growth Conditions

The functional male sterile line S12 and fertile line F142 were provided and grown at the Institute of Vegetables and Flowers, Chongqing Academy of Agricultural Sciences (Chongqing, China) from 2017 to 2019. The male fertile line was an advanced-generation inbred line. The functional male sterile line was obtained from the continuous backcross of male sterile plant in progenies F2 of interspecific hybrid (Tian et al., 2001). The eggplant seeds were sterilized and sown in trays. Then, the seedlings were transferred and grown under normal conditions. Selected anthers in flower buds with open petals about 10 am were immediately frozen in liquid nitrogen and stored at −80°C until they were used for further analysis. Three biological replicates per sample were used for sequencing.

### RNA Extraction, Library Construction, and RNA-Seq

The total RNA of each sample was extracted from the anther on the day of flowering of eggplant according to the instruction manual of the TRlzol Reagent (Life Technologies, Carlsbad, CA, United States). Each anther sample was taken from five eggplant flowers. RNA integrity and concentration were examined using Agilent 2100 Bioanalyzer (Agilent Technologies, Inc., Santa Clara, CA, United States). The mRNA was isolated by NEBNext Poly (A) mRNA Magnetic Isolation Module (NEB, E7490). The cDNA library was constructed following the instructions of the manufacturer of NEBNext Ultra RNA Library Prep Kit for Illumina (NEB, E7530) and NEBNext Multiplex Oligos for Illumina (NEB, E7500). In brief, the enriched mRNA was fragmented into ~200 nt RNA inserts, which were used to synthesize the first-strand cDNA and the second cDNA. End-repair/dA-tail and adaptor ligation was performed on the double-stranded DNA. Suitable fragments were isolated by Agencourt AMPure XP beads (Beckman Coulter, Inc., Brea, CA, United States) and enriched by PCR amplification. Finally, constructed cDNA libraries of the eggplant were sequenced on a flow cell using an Illumina HiSeq™ (Illumina, San Diego, CA, United States) sequencing platform. The RNA-seq reads have been deposited in the NCBI Short Read Archive and are accessible under PRJNA746400.

### Transcriptome Analysis Using Reference Genome-Based Reads Mapping

Low-quality reads, such as only adaptor and unknown nucleotides >5%, or Q20 <20% (percentage of sequences with sequencing error rates <1%), were removed by Perl script. The clean reads that were filtered from the raw reads were mapped to the eggplant genome (SME_r2.5.1) (Hideki et al., 2014) using Tophat2 software2 (version2.1.0) (Kim et al., 2013). The aligned records from the aligners in BAM/SAM format were further examined to remove potential duplicate molecules. Gene expression levels were estimated using FPKM values (fragments per kilobase of exon per million fragments mapped) by the Cufflinks software.

### Sequence Annotation

Genes were compared against various protein databases by BLASTX, such as the National Center for Biotechnology Information (NCBI) non-redundant protein (Nr) database, and the Swiss-Prot database, with a cut-off E-value of 10^−5^. Furthermore, genes were searched against the NCBI non-redundant nucleotide sequence (Nt) database using BLASTn with a cut-off E-value of 10^−5^. Genes were retrieved based on the best BLAST hit (highest score) along with their protein functional annotation.

To annotate the gene with GO terms, the Nr BLAST results were imported into the Blast2 GO program. GO annotations for the genes were obtained by Blast2GO. This analysis mapped all of the annotated genes to GO terms in the database and counted the number of genes associated with each term. Perl script was then used to plot GO functional classification for the unigenes with a GO term hit to view the distribution of gene functions. The obtained annotation was enriched and refined using TopGo (R package). The gene sequences were also aligned to the Clusters of Orthologous Group (COG) database to predict and classify functions. KEGG pathways were assigned to the assembled sequences by the Perl script.

### Identification of Differential Gene Expression

DESeq2 and Q-value were employed and used to evaluate differential gene expression between F142 and S12. After that, gene abundance differences between those samples were calculated based on the ratio of the 2 FPKM values. In order to compute the significance of the differences, the false discovery rate (FDR) control method was used to identify the threshold of the *P*-value in multiple tests. Here, only genes with an absolute value of log2 ratio ≥2 and an FDR significance score of <0.01 were used for subsequent analysis.

### Gene Ontology and Kyoto Encyclopedia of Genes and Genome Enrichment Analysis of DEGs

Gene ontology enrichment analysis of DEGs was implemented by the GOseq R package. GO terms with corrected *P*-values <0.05 were considered to be significantly enriched by differentially expressed genes (Young et al., 2010). The KOBAS software (Mao et al., 2005) was used to test the statistical enrichment of DEGs in KEGG pathways.

### Hormone Extraction and Determination

High-performance liquid chromatography was performed using Shimadzu LC-60A (Shimadzu, Kyoto, Japan. The chromatographic conditions were as follows: mobile phase was methanol 0.8% glacial acetic acid solution = 55/45, column temperature was 30°C, flow rate was 0.8 mL/min, detection wavelength was 254 nm, and injection volume was 10 μL. Each sample was tested three times and averaged. All data were analyzed by ANOVA, and the differences were compared by Duncan's multiple range test.

### Gene Cloning and Real-Time Quantitative Reverse Transcription-PCR

The full length of *SmDAD1* (Smechr0500450), *SmLOX* (Smechr0800437), *SmJAR1* (Smechr0101378), *SmCOI1* (Smechr0500307), and *SmJAZ1* (Smechr1200204) was cloned using homologous cloning technology. Tomato and potato sequences closely related to eggplant were obtained from the NCBI database. After the two Blast, the software Primer 5.0 was used to design gene-specific primers (GSPs). RNA was extracted from eggplant anthers and reverse transcribed into cDNA. Then, RT-PCR was performed using the extracted RNA as a template to obtain ORFs of *SmDAD1, SmLOX, SmJAR1, SmCOI1*, and *SmJAZ1* genes.

Quantitative reverse transcription-PCR was performed as previously described. The primers used to test the transcript levels of all the genes were shown in Supplementary Table 1, using *GAPDH* as the internal reference. The qRT-PCR mixtures contained 2 μL primers, 2 μL cDNA, 10 μL SsoFast™EvaGreen®Supermix (Bio-Rad, Hercules, CA, United States) and distilled water to a final volume of 20 μL. The reaction conditions were as follows: 95°C for 30 s, 95°C for 5 s, 59°C for 30 s, and 65°C for 5 s (39 cycles). The fold changes were calculated using the 2^−Δ*ΔCt*^ method. Each sample was repeated three times for qRT-PCR detection. And the fold changes were calculated using the 2^−Δ*ΔCt*^ method.

### Transactivation Test in Yeast

*SmDAD1, SmLOX, SmJAR1, SmCOI1*, and *SmJAZ1* were separately sub-cloned into the activation domain of pGADT7 and pGBKT7 using the *Bam*HI and *Xho*I sites and then ligated into pGADT7 or pGBKT7 to construct recombinant plasmids. First, gene-pGBKT7 recombinant plasmids were transformed into Y2H (Clontech) using the PEG/LiAC method. The transformed strains were screened on synthetic dropout (SD medium) lacking tryptophan (Trp; SD/-Trp) for selection of positive clones. Subsequently, positive clones were transferred to SD medium supplemented with X-α-gal(SD/-Trp/X-α-gal). The trans-acting activity was assessed based on the blue colonies that grew on the SD/-Trp/X-α-gal medium.

### Yeast Two-Hybrid Assay

Yeast two-hybrid assays were performed based on the instructions of the manufacturer (Clontech, Palo Alto, CA, United States). The gene-pGADT7 and gene-pGBKT7 recombinant plasmids were co-transformed into yeast strainY2HGold cells as described above, which were then added to SD medium lacking leucine (Leu) and tryptophan (Trp) (DDO; SD/-Leu/-Trp). The potential physical interactions between proteins were evaluated by screening the yeast transformants on QDO/X-α-gal/AbA medium [SD medium lacking Leu, Trp, adenine (Ade), and histidine (His) but supplemented with X-a-gal and aureobasidin A (AbA)].

### Pull-Down Assay

*SmDAD1* was sub-cloned into the pET32a(+) vector, while *SmLOX, SmJAR1, SmCOI1*, and *SmJAZ1* were cloned into the pGEX-4T-1 vector. Then, the plasmids were transformed into *Escherichia coli* Rosetta (DE3) competent cells, and 1 mM isopropyl β-D-thiogalactoside was added before incubation at 37°C for 3.5 h. The SmDAD1-HIS protein was purified using BeaverBeads IDA-Nickel Kit-10 (Beaver, Beijing, China). The SmLOX-GST, SmJAR1-GST, SmCOI1-GST, and SmJAZ1-GST proteins were purified by BeaverBeads GSH (Beaver). Protein–protein interactions were detected by sodium dodecyl sulfate-polyacrylamide gel electrophoresis (SDS-PAGE).

## Results

### Morphological Comparison of F142 and S12

**Figure 1:**
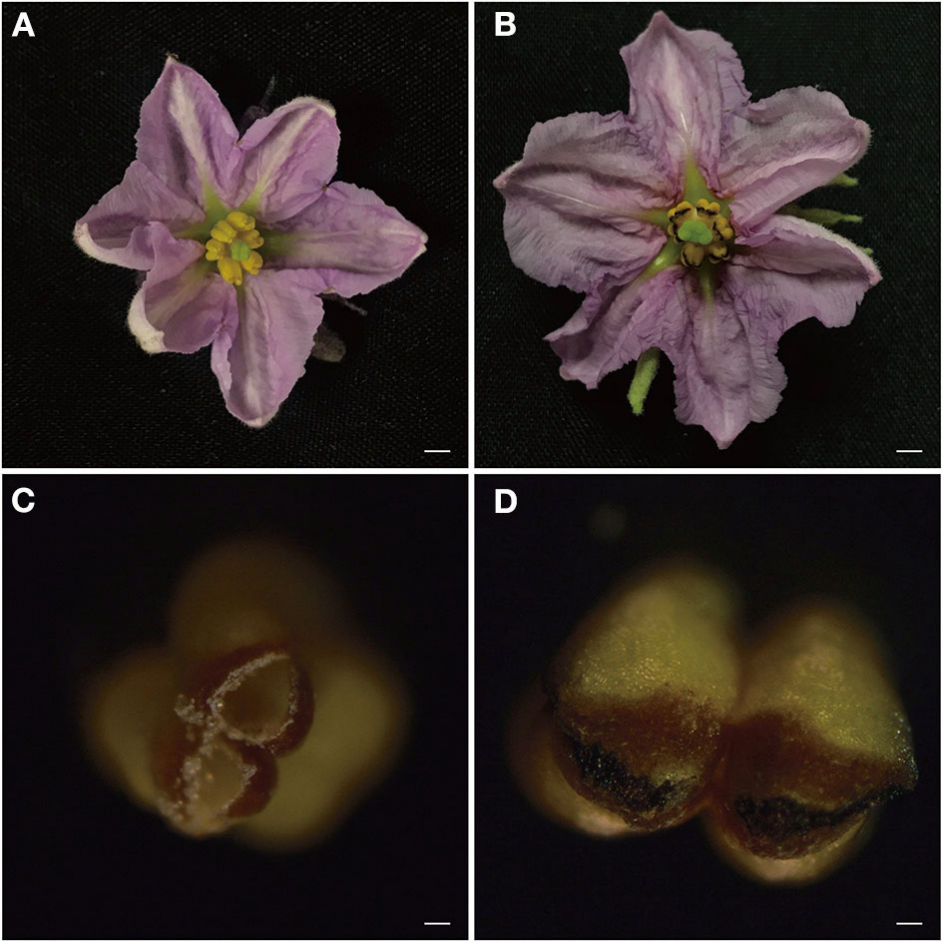
Morphological analysis of *Solanum melongena* L. F142 and S12 flowers and anthers on the day of flowering. **(A,B)** The flower of F142 and S12. Bars = 5 mm. **(C,D)** The anther of F142 and S12. Bars = 100 μm.

Male sterility is an important tool for leveraging eggplant heterosis. Anther indehiscence is the main form of functional male sterility. By morphological analysis, we observed that anthers were indehiscent in S12 relative to F142 (Figure 1). On the day of flowering, in F142, the anthers presented small holes to release pollens, whereas in S12 the anthers were tightly closed without dehiscence and pollen releasing.

### Transcriptome Assembly

To detect potential molecular differences between eggplant accessions S12 and F142, we performed transcriptome sequencing on anther tissue on the day of flowering. The genome-directed stratagem Trinity was used to assemble transcriptome sequences and align single RNA-seq library data with the *S. melongena* genome (SME_r2.5.1). As a result, the mean of clean reads from three biological repeats per sample was more than 20 million, the mean of mapped reads and uniquely mapped reads were far great than 10 million, and the highest localization ration of each library was roughly 50% in the *S. melongena* genome (Table 1; Supplementary Table 2). After being assembled, the average length of the contig was 1,204 bp, while for the contig of N50 the average length was 1,538 bp. We found that the average GC content in the libraries reached 40–50% (Supplementary Tables 2, 3). When redundant and short reads were removed, we obtained about 257,800 transcript assembly contigs (TAC) >100 bp (Supplementary Table 2). Overall, the abundant transcriptome data were enough for further analysis.

**Table 1:** Statistical analysis of RNA-seq reads mapped to the ancestor genome.

**Samples**	**Total reads**	**Mapped reads**	**Uniq mapped reads**	**Multiple map reads**	**Clean reads**	**GC content**	**%≥Q30**
F142	42,791,674.67	39,368,088	38,019,098	1,348,989	21,395,837	42.68%	94.41%
S12	43,133,323.33	39,694,750	38,266,857	1,427,894	21,566,662	42.93%	94.70%

### Functional Classification of the DEGs by GO and KEGG Pathway Analysis

We identified 2,670 DEGs in S12 vs. F142 (Supplementary Table 4; Supplementary Figure 2), including 1,928 upregulated and 742 downregulated DEGs (Supplementary Figure 2). We constructed a heat map representing the differential expression of the 2,670 DEGs (Supplementary Figure 5) in S12 vs. F142. We detected dynamic changes in the transcriptomes during anther development in S12 (anther indehiscence). In the heat map, the original gene expression data were transformed into log2 fold change values (Supplementary Table 4). Further cluster analysis revealed significant differences in gene expression between S12 and F142.

**Figure 2:**
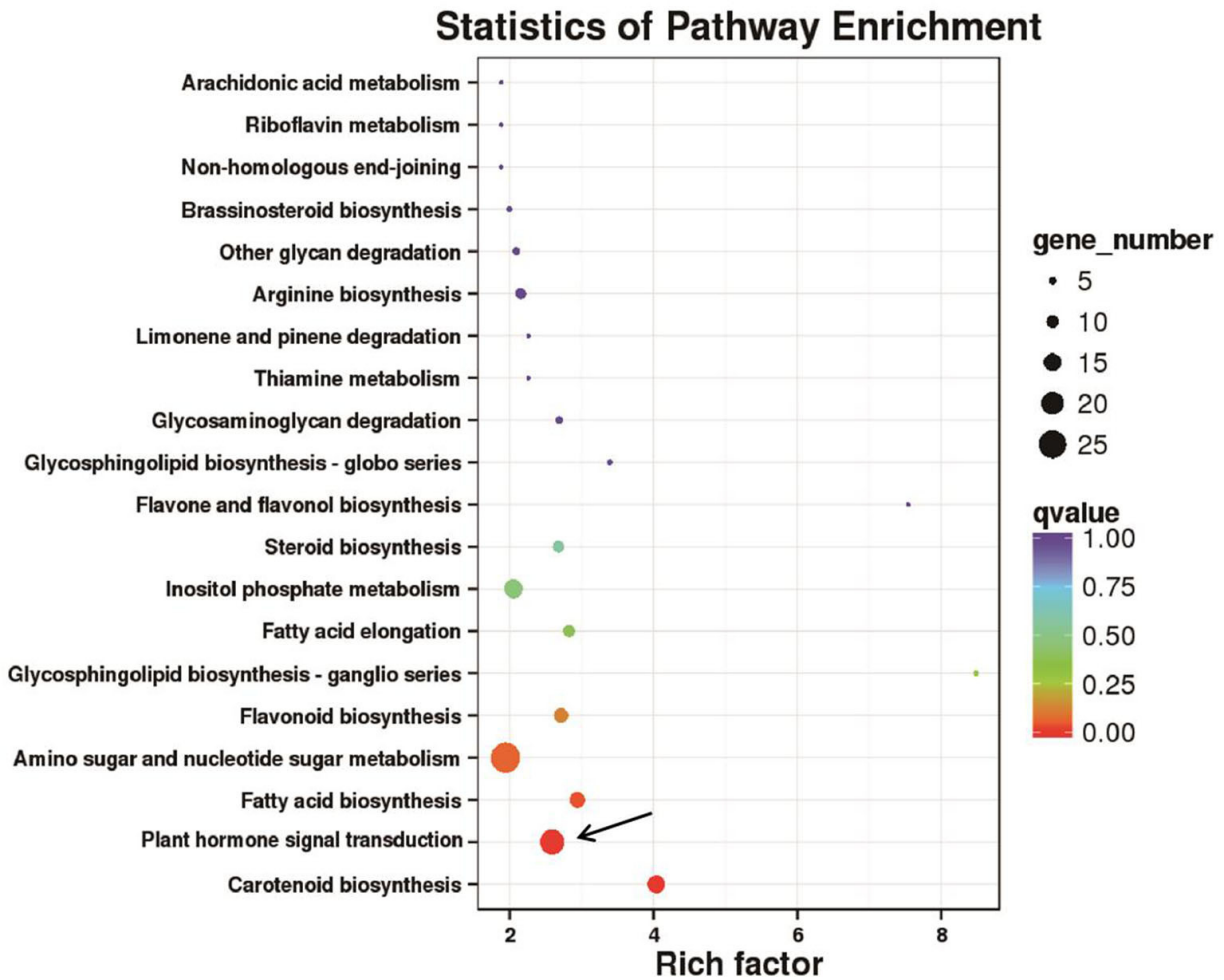
Kyoto Encyclopedia of Genes and Genomes (KEGG) pathway enrichment analysis of 2,670 differentially expressed genes (DEGs). The pathway of plant hormone signal transduction was mainly enriched (black arrows).

To characterize the DEGs in detail, we performed GO analysis to uncover their putative functions. We constructed histograms based on the categories of DEGs in anther indehiscent eggplant (S12), such as biological processes, molecular functions, and cellular compartments (Supplementary Figure 3). Metabolic process was the major group in the biological processes category, namely, GO terms cellular, single-organism, response to stimulus, and biological regulation. The molecular functions category contained at least 882 DEGs involved in nucleic acid binding transcription factor activity and more than 888 DEGs involved in signal transducer activity, transcription factor activity, and protein binding. Most of the DEGs were present in the top three groups, such as the cell, organelle, and membrane (Supplementary Figure 4). We performed KEGG pathway analysis to categorize all annotated genes. Most of the 2,670 DEGs were categorized into six pathways (Supplementary Figure 4). The most highly enriched biological processes in the anther indehiscent eggplant were plant hormone signal transduction, protein processing in endoplasmic reticulum, amino sugar and nucleotide sugar metabolism, biosynthesis of amino acids, carbon metabolism, and plant–pathogen interactions (Figure 2; Supplementary Figure 4).

### Enrichment Analysis of DEGs in Hormone Signal Transduction Pathways

**Figure 3:**
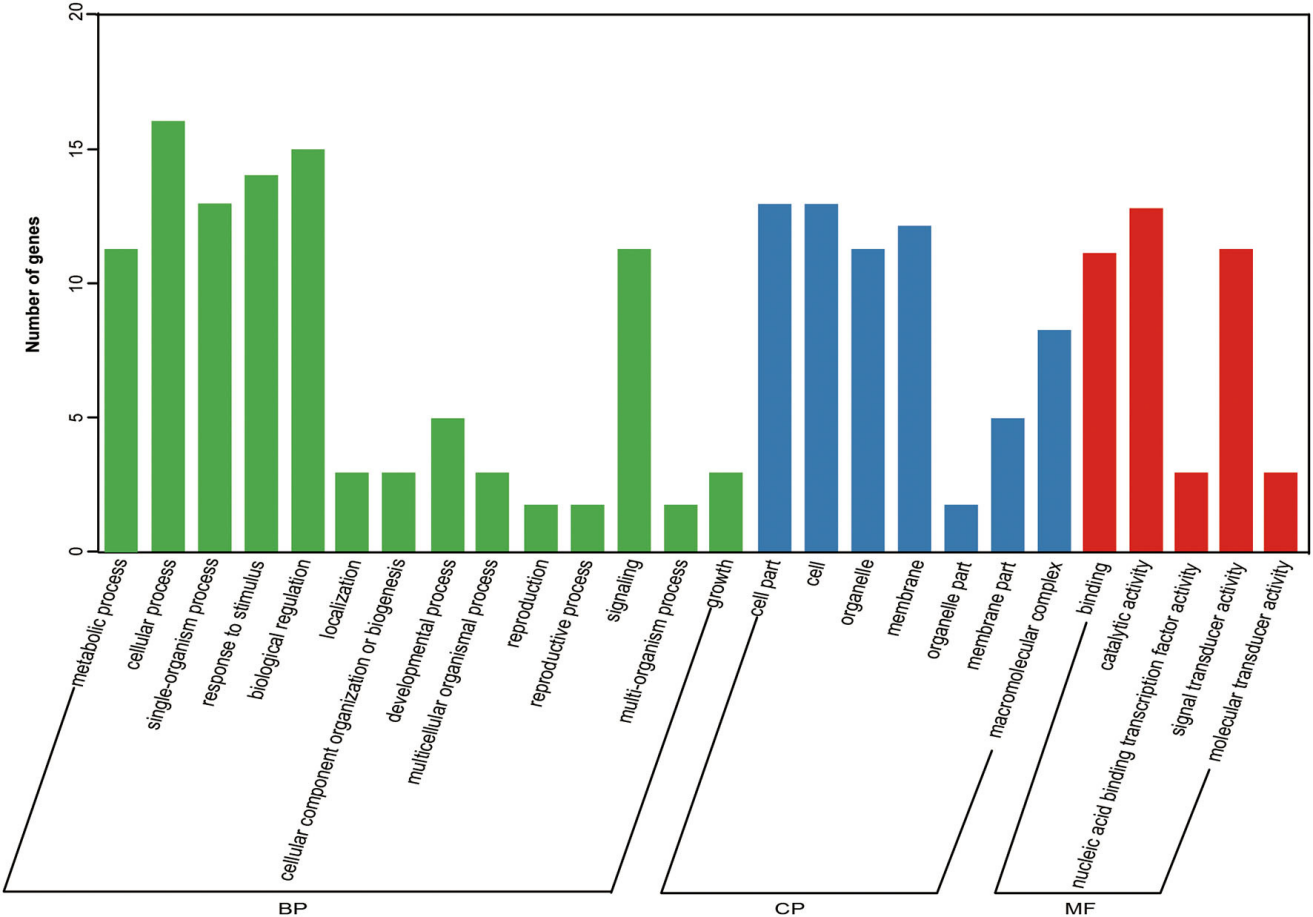
Gene ontology (GO) enrichment analysis of 31 hormone DEGs. BP, biological processes; CP, cellular compartments; MF, molecular functions.

**Figure 4:**
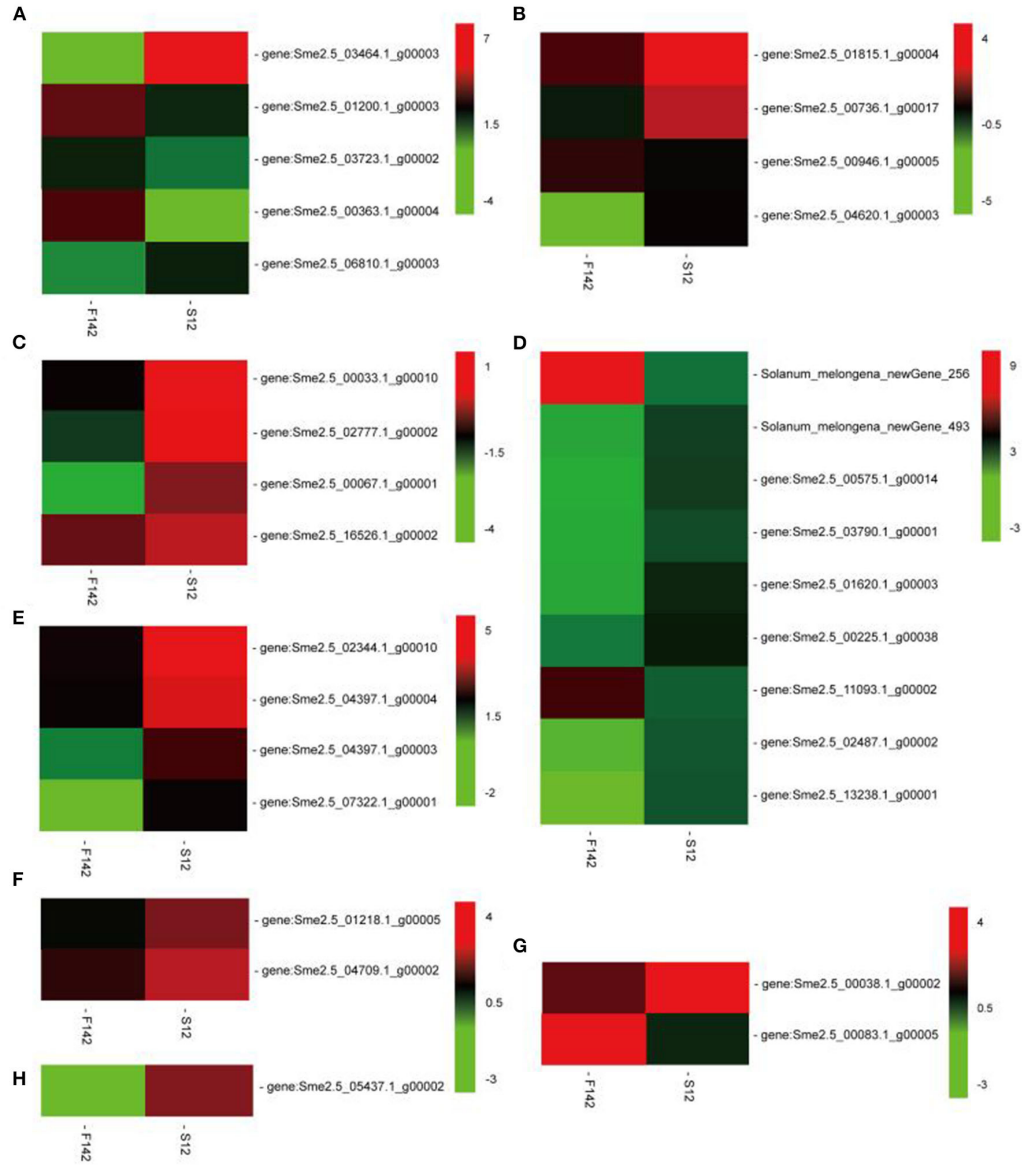
Expression changes in the genes involved in the **(A)** jasmonic acid, **(B)** ethylene, **(C)** auxin, **(D)** other hormones, **(E)** crassinolide, **(F)** cytokinin, **(G)** gibberellin, and **(H)** abscisic acid signaling pathways in F142 and S12.

We performed KEGG pathway analysis to investigate the major regulatory pathways of the DEGs. The DEGs in S12 vs. F142 were mainly enriched in the pathways inositol phosphate metabolism (16 DEGs), plant hormone signal transduction (31 DEGs), flavonoid biosynthesis (12 DEGs), amino sugar and nucleotide sugar metabolism (39 DEGs), and fatty acid biosynthesis (25 DEGs) (Figures 2, 3). To investigate the hormonal control of anther indehiscence in more detail, we analyzed the expression levels of key DEGs in the JA, IAA, GA, ABA, cytokinin (CTK), ethylene (ETH), and brassinosteroid (BR) signaling pathways (Figure 4; Table 2). Five key genes were identified in the JA signaling pathway, namely, *SmDAD1, SmLOX, SmCOI1, SmJAZ1*, and *SmJAR*-like, of which two genes were significantly upregulated and three genes were downregulated in S12 vs. F142. One key gene in the CTK signaling pathway and two genes in the ABA signaling pathway were differentially expressed. One gene in the GA signaling pathway was significantly upregulated, and the other was significantly downregulated. Four key genes in the IAA signaling pathway were differentially expressed. Four key genes in the ETH signaling pathway were also differentially expressed, which were all significantly upregulated. Four genes in the BR pathway were significantly upregulated as well. Finally, nine genes in other hormone signaling pathways were differentially expressed, including two that were significantly downregulated in indehiscent accession (Figure 4).

**Table 2:** Differentially expressed genes (DEGs) involved in hormone signaling pathways.

**Gene_Id**	**Function description**	**Up_Down**
**Jasmonate acid**		
Sme2.5_03464.1_g00003	Jasmonate ZIM-domain protein 1	Up
Sme2.5_01200.1_g00003	Defective in anther dehiscence1	Down
Sme2.5_03723.1_g00002	Coronatine-insensitive 1	Down
Sme2.5_00363.1_g00004	Lipoxygenase	Down
Sme2.5_06810.1_g00003	Jasmonic acid-amido synthetase JAR1	Up
**Auxin**		
Sme2.5_01815.1_g00004	Auxin influx carrier (LAX family), LAX1	Up
Sme2.5_00736.1_g00017	Auxin responsive GH3 gene family, indole-3-acetic acid-amido synthetase GH3.1	Up
Sme2.5_00946.1_g00005	Auxin-responsive protein IAA26-like	Down
Sme2.5_04620.1_g00003	Auxin responsive GH3 gene family, indole-3-acetic acid-amido synthetase GH3.6	Up
**Brassinosteroid**		
Sme2.5_00033.1_g00010	Brassinosteroid resistant 1-like	Up
Sme2.5_02777.1_g00002	Brassinosteroid insensitive 2, shaggy-related protein kinase eta-like	Up
Sme2.5_00067.1_g00001	Brassinosteroid insensitive 1, BRI1	Up
Sme2.5_16526.1_g00002	Brassinosteroid resistant 1, BES1/BZR1 homolog protein 2-like	Up
**Ethylene**		
Sme2.5_02344.1_g00010	Ethylene receptor 2-like	Up
Sme2.5_04397.1_g00003	Ethylene-insensitive protein 3, EIN3	Up
Sme2.5_04397.1_g00004	Ethylene-insensitive protein 3, EIN3-like	Up
Sme2.5_07322.1_g00001	Ethylene receptor 1, ETR1	Up
**Cytokinin**		
Sme2.5_05437.1_g00002	Histidine kinase 2-like isoform X1 (cytokinin receptor)	Up
**Abscisic Acid**		
Sme2.5_01218.1_g00005	Abscisic acid-insensitive responsive element binding factor, ABA 5-like protein 7	Up
Sme2.5_04709.1_g00002	ABA responsive element binding factor, G-box-binding factor 4	Up
**Gibberellin**		
Sme2.5_00083.1_g00005	Gibberellin receptor GID1B-like	Down
Sme2.5_00038.1_g00002	F-box protein GID2	Up
**Other hormone**		
Sme_newGene_256	SAUR family protein, uncharacterized LOC107006819	Down
Sme_newGene_493	Myb-like DNA-binding domain, two-component response regulator ARR14	Up
Sme2.5_00575.1_g00014	Protein phosphatase 2C 6-like	Up
Sme2.5_03790.1_g00001	Myb-like DNA-binding domain, Two-component response regulator ARR18-like	Up
Sme2.5_01620.1_g00003	Serine/threonine-protein kinase SRK2	Up
Sme2.5_00225.1_g00038	Somatic embryogenesis receptor kinase 3B precursor, SERK3B	Up
Sme2.5_11093.1_g00002	SAUR family protein, uncharacterized protein LOC104880086	Down
Sme2.5_02487.1_g00002	Serine/threonine-protein kinase, SAPK3	Up
Sme2.5_13238.1_g00001	Two-component response regulator ARR17-like	Up

### Endogenous Hormone Measurements and Validation of the Expression Patterns of Several Key Genes

**Figure 5:**
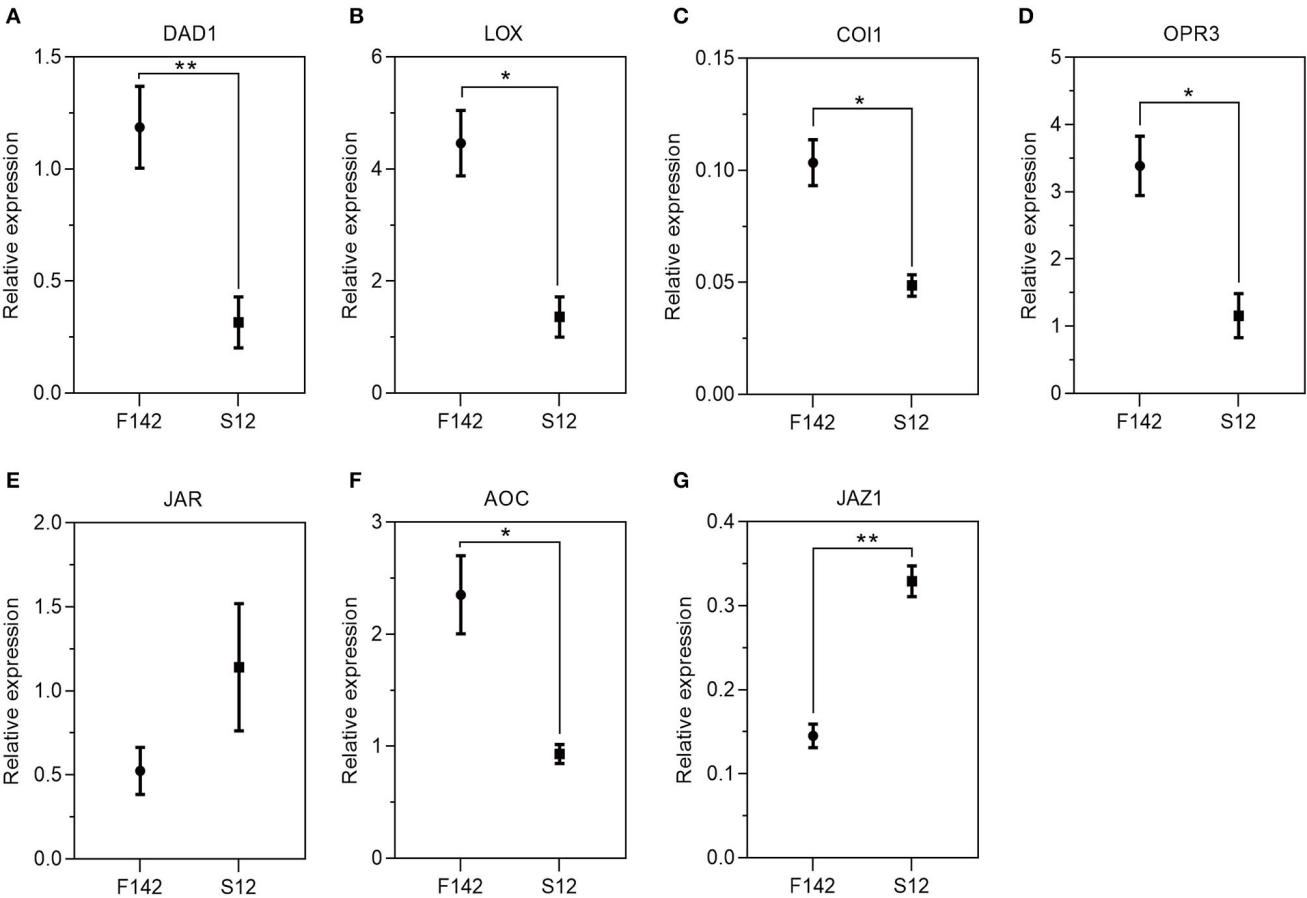
The transcript accumulation of **(A)** *SmDAD1*, **(B)** *SmLOX*, **(C)** *SmCOI1*, **(D)** *SmOPR3*, **(E)** *SmJAR1*, **(F)** *SmAOC*, and **(G)** *SmJAZ1*. Total RNA was extracted in anthers and used for RT-q PCR analyses. Three biological replicates (each including three technical repeats) were assessed. Paired *t*-tests, *p < 0.05, **p < 0.01.

**Figure 6:**
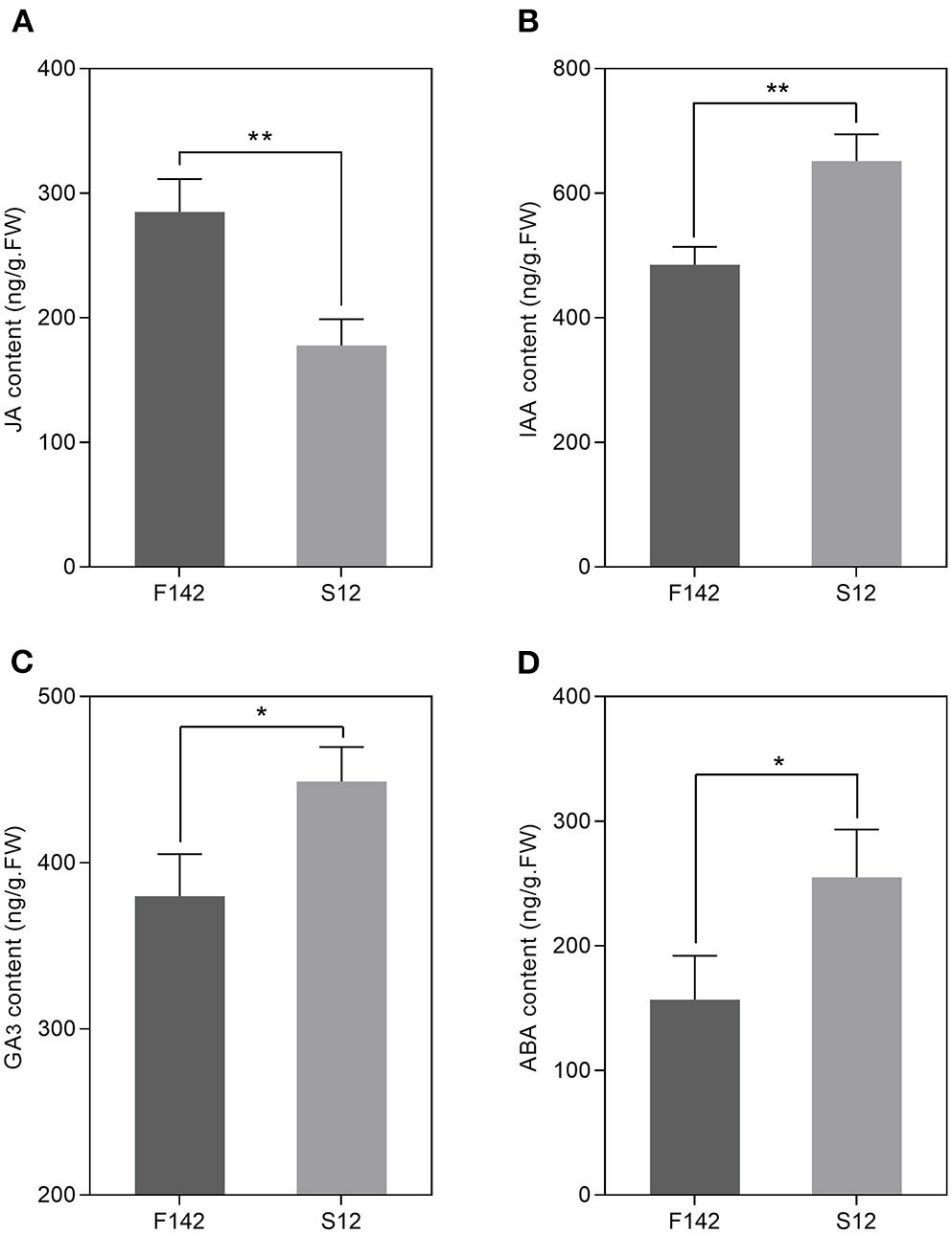
Changes in JA, IAA, GA_3_, and ABA concentrations in F142 and S12. **(A)** JA concentration. **(B)** IAA concentration. **(C)** GA_3_ concentration. **(D)** ABA concentration. Three independent biological replicates were used. Three biological replicates were assessed. Paired *t*-tests, *p < 0.05, **p < 0.01.

We collected independent anther samples from the plants and performed qRT-PCR analysis to validate the expression levels of several key JA-related genes. In total, we measured the expression levels of seven JA-related unigenes via qRT-PCR. *SmJAZ1* and *SmJAR1* were significantly upregulated and *SmDAD1, SmLOX, SmOPR3, SmAOC*, and *SmCOI1* were significantly downregulated in S12. The expression patterns of these genes corresponded well with the FPKM values obtained by RNA-seq (Figure 5), suggesting that the expression patterns of most unigenes were consistent between the two methods. Finally, we measured JA, IAA, GA_3_, and ABA levels in S12 and F142 (Figure 6). Compared with F142, the levels of IAA, GA_3_ and ABA in S12 were significantly increased, while JA level was significantly decreased. These findings suggested that plant hormones play an important role in anther dehiscence, and the decrease of JA may cause anther indehiscence in eggplant.

### Interaction of SmDAD1 With SmLOX, SmJAR1, SmCOI1, and SmJAZ1

We first analyzed the trans-acting activity of SmDAD1, SmLOX, SmJAR1, SmCOI1, and SmJAZ1 in a yeast system. The yeast cells containing gene-pGBKT7 recombinant plasmids grew well and appeared white when screened on the selective medium (SD/-Trp) supplemented with X-α-gal (Supplementary Figure 6). These results reflected the *trans*-acting activity of SmDAD1-BD, SmLOX-BD, SmJAR1-BD, SmCOI1-BD, and SmJAZ1-BD that was similar to that of BD.

**Figure 7:**
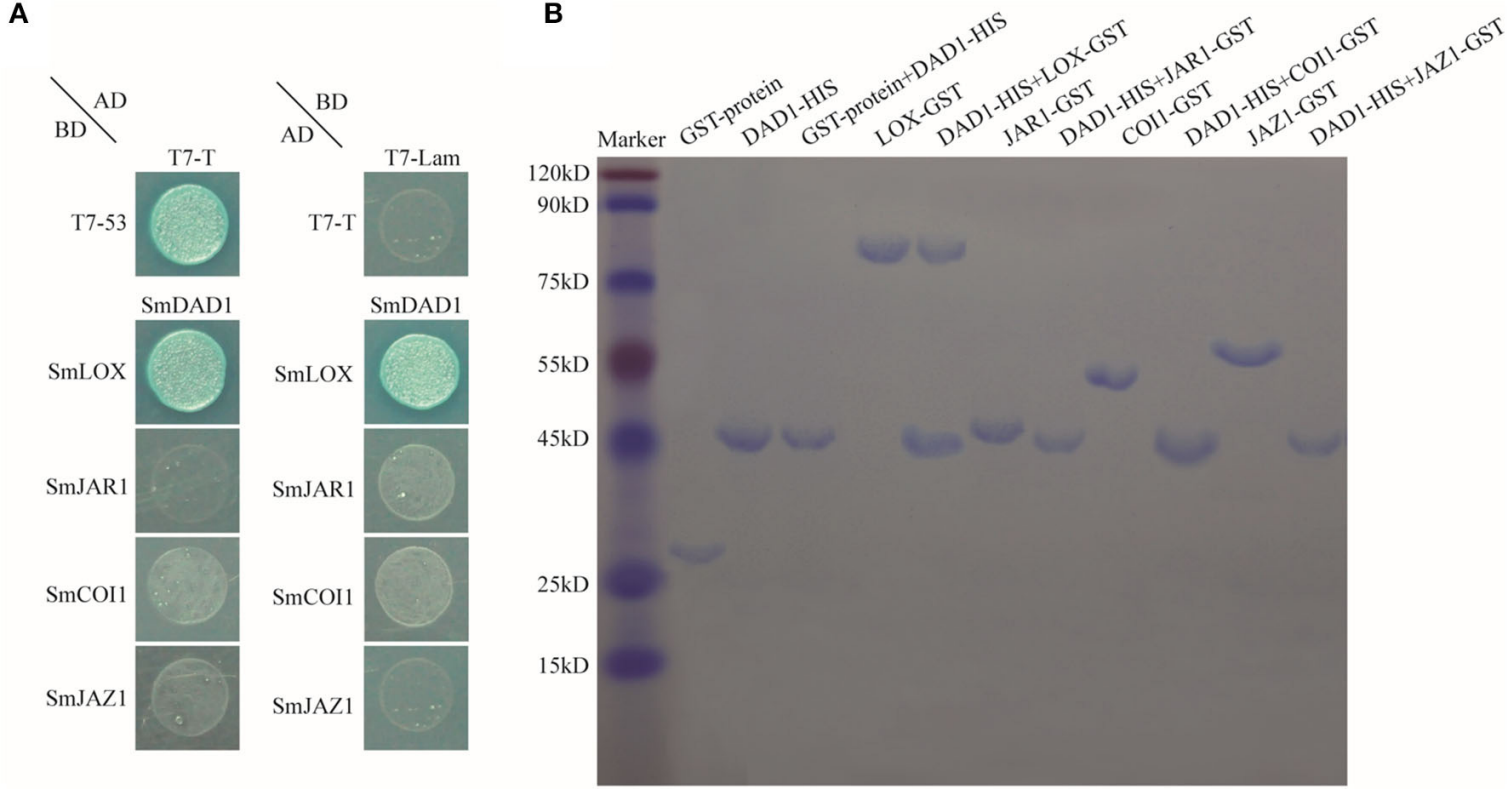
Protein interactions of SmDAD1 with SmLOX, SmJAR1, SmCOI1, and SmJAZ1. **(A)** Detecting interactions of SmDADA1 with SmLOX, SmJAR1, SmCOI1, and SmJAZ1 by yeast two-hybrid assay. Transformed yeast cells were plated on SD/-Ade/-His/-Leu/-Trp/X-a-Gal medium to grow at 30°C for 3–5 days. pGBKT7-T53 (T7-T53) combined with pGADT7-T (T7-T) was used as positive controls, and pGBKT7-lam (T7-lam) combined with pGADT7-T (T7-T) was used as negative controls. **(B)** Examining the interactions of SmDAD1 with the other proteins by Pull-down. The HIS-tagged SmDAD1 protein was generated by cloning into the pET32a (+) vector (19 kDa). The GST-tagged proteins of SmLOX, SmJAR1, SmCOI1, and SmJAZ1 were generated by cloning into the pGEX-4T-1 vector (26 kDa). Bound proteins were eluted and stained with Coomassie Brilliant Blue 250 and then separated by 12.5% SDS-PAGE.

Subsequently, we performed yeast two-hybrid assays to detect the interactions of SmDAD1 protein with SmLOX, SmJAR1, SmCOI1, and SmJAZ1 (Figure 7A). SmDAD1 directly interacted with SmLOX but not with SmJAR1, SmCOI1, or SmJAZ1 (Figure 7A). We also performed pull-down assays to determine whether SmDAD1 interacts with SmJAR1, SmCOI1, and SmJAZ1 (Figure 7B). SmLOX-GST was pulled down by SmDAD1-HIS but SmJAR1-GST, SmCOI1-GST, and SmJAZ1-GST were not. Therefore, our yeast two-hybrid results were replicated in the pull-down assays (Figure 7).

## Discussion

Transcriptome analysis facilitates the comprehensive investigation of altered gene expression patterns in genetic variants and provides insights into the molecular basis of specific biological processes (Liu et al., 2019). Transcriptome sequencing provides a systematic approach for studying gene expression patterns and network interactions underlying various processes in plants. In this study, to explore the molecular mechanism underlying anther dehiscence in eggplant, we generated a high-quality transcriptome dataset from dehiscence (F142) and indehiscence (S12) anthers. The most highly enriched biological processes among the DEGs in the indehiscent anthers were plant hormone signal transduction, protein processing in endoplasmic reticulum, amino sugar and nucleotide sugar metabolism, biosynthesis of amino acids, carbon, metabolism, and plant–pathogen interaction. In addition, by comparing the transcripts of F142 and two male-sterile lines (S12 and S13), the differentially expressed genes in the sterile line were mainly enriched in “metabolic process,” “catalytic activity,” “biosynthesis of amino acids,” and “carbon metabolism” (Yuan et al., 2020).

Thirty-one DEGs were identified (Table 2) involved in hormone signal transduction pathways, such as JA, IAA, GA, ABA, CTK, ETH, and BR. Phytohormones play essential roles in regulating plant growth and development as well as plant fertility. The accumulation or deficiency of auxin in plants is related to the occurrence of male sterility. The IAA content in a cytoplasmic male sterile rapeseed line was consistent with that of normal plants under low-temperature conditions. However, the IAA content of the sterile line increased with increasing temperature, whereas no change in IAA levels was detected in fertile plants (Singh et al., 1992). This phenomenon was also observed in tomato mutants (Amit and Sawhney, 1993). Horner suggested that auxin accumulation caused male sterility in crops, as high IAA levels in pepper induced the production of ETH, which induced male sterility (Horner, 1977). In addition, during pollen abortion, IAA levels were lower in two types of sterile wheat than in fertile anthers (Li et al., 1976). This phenomenon was also observed in rice (Xu et al., 1990), citrus (Tian et al., 1998), and mustard (Kojima, 1997).

Moreover, CTK levels were lower in cytoplasmic male sterile barley lines than in their maintainer lines (Chen et al., 1995). The excess ABA in the leaves and anthers of cytoplasmic male sterile cabbage lines might be related to the occurrence of microspore abortion (Shi and Hou, 2004). Liu found that in male sterile wheat lines (induced by GENESIS), after induction, ETH levels were significantly higher in sterile lines than in fertile lines during the mononuclear, dinuclear, and trinuclear stages of anther development (Liu et al., 2003). The rate of infertility increased with increasing induction, and the rate of infertility and the ETH release rate also increased. Finally, wild-type *Arabidopsis* plants treated with GA and double mutants in the GA signaling repressors RGA and GAI exhibited loss of fertility (Dill and Sun, 2001).

Plant development and responses to environmental signals are coordinated by complex multicomponent signaling networks. JA, a phytohormone derived from fatty acids, is an important component of this regulatory system. It participates in all stages of plant growth and development and also regulates anther dehiscence (Ching-Fang et al., 2014; Xiao et al., 2014). In this study, the transcript levels of JA biosynthesis genes were lower in anther indehiscent plants than in plants with normal anther development. The JA content was also significantly lower in these plants than in fertile eggplant. This observation, which is consistent with the results of transcriptome sequencing, confirms the notion that JA is an essential factor affecting anther dehiscence. This finding validates the results of previous studies (Sanders, 2000; Stintzi and Browse, 2000; Malek et al., 2002). For example, mutations in genes involved in JA biosynthesis typically caused delayed or failed anther dehiscence, such as *DAD1* (Ishiguro et al., 2001; Qin et al., 2011) and *OPR3* (Stintzi and Browse, 2000; Chini et al., 2018).

Based on the current transcriptome data for genes in the JA biosynthesis pathway, we propose that feedback regulation of JA signaling in anther-indehiscent eggplant alters the expression patterns of genes at the mRNA level during anther development (Sanders, 2000; Zhao and Ma, 2000; Hong, 2005). In this study, we identified five genes in the JA pathway (*SmDAD1, SmLOX, SmCOI1, SmJAZ1*, and *SmJAR1*) that were differentially expressed in the S12 vs. F142 eggplant. *SmDAD1, SmLOX*, and *SmCOI1* were clearly downregulated in S12, whereas *SmJAZ1* and *SmJAR1* were upregulated in S12 vs. F142. *SmDAD1* is crucial for JA biosynthesis. *Arabidopsis DAD1* (*At2g44810*) encodes the first chloroplastic lipase identified. This enzyme is involved in supplying α-linolenic acid for the JA-biosynthetic pathway (Ishiguro et al., 2001). Mutations in *DAD1* reduced JA levels in flower buds, causing a delay in their development, failed anther dehiscence during flower opening, and lack of pollen grain maturation (Ishiguro et al., 2001). In the *Arabidopsis coi1, opr3*, and *dad1* mutants, the anthers failed to crack and the filaments were short; however, these phenotypes were significantly altered by the external application of JA (Stintzi and Browse, 2000; An et al., 2018).

The JA pathway involves a series of gene-encoded hormone-related factors involved in anther dehiscence (Grunewald et al., 2009; Xiao et al., 2014; Chini et al., 2018). Previous gene expression analysis has demonstrated that overexpressing *AtOPR3* selectively affected the expression of various genes of the endogenous jasmonate system, while the expression of other genes remained unaltered. Transgenic wheat plants with high *AtOPR3* expression levels exhibited notably altered plant growth and development, including delayed germination, slower growth, and anther indehiscence (Pigolev et al., 2018). These findings indicate that these plant phenotypes are regulated by direct or indirect interactions of these genes.

Melotto et al. (2008) demonstrated that the physical interaction between COI1 and JAZ proteins could be effectively promoted by treatment with biologically active jasmonates (JA-Ile) (Singh et al., 1992; Melotto et al., 2008). In addition, AtMYC2 interacts with JAZs (Wasternack, 2017; Chini et al., 2018). Here, we demonstrated that SmDAD1 interacts with SmLOX1 both *in vitro* and *in vivo*. However, how these proteins regulate anther dehiscence remains unclear and should be addressed in future studies.

## Conclusions

The outcomes of this study revealed that 31 DEGs related to hormone biosynthesis were identified by transcriptome between the anther-dehiscent eggplant (F142) and the anther-indehiscent eggplant (S12). Among them, the JA level of S12 was significantly lower than that of F142. The study on protein–protein interaction confirmed the direct interaction between SmDAD1 and SmLOX. Therefore, JA was confirmed to play an important role in anther dehiscence of eggplant.

## Data Availability Statement

The RNA-seq reads presented in the study are publicly available. This data can be found at the NCBI Short Read Archive (PRJNA746400).

## Author Contributions

ZW and YN designed the research. CY and SZ performed the molecular biology experiments. QT, DW, and ST carried out the bioinformatics analysis. ZW, CY, and SZ analyzed the data and wrote the study manuscript. All the authors approved the final version of the manuscript.

## Conflict of Interest

The authors declare that the research was conducted in the absence of any commercial or financial relationships that could be construed as a potential conflict of interest.
