# Supplementary material for: Screening and Interaction Analysis Identify Genes Related to Anther Dehiscence in Solanum melongena L
Source: Front Plant Sci. 2021 Jul 22;12:648193. doi: 10.3389/fpls.2021.648193 (PMC8341306; doi:10.3389/fpls.2021.648193)
Supplement: Supplementary file 1 [file Data_Sheet_1.docx]

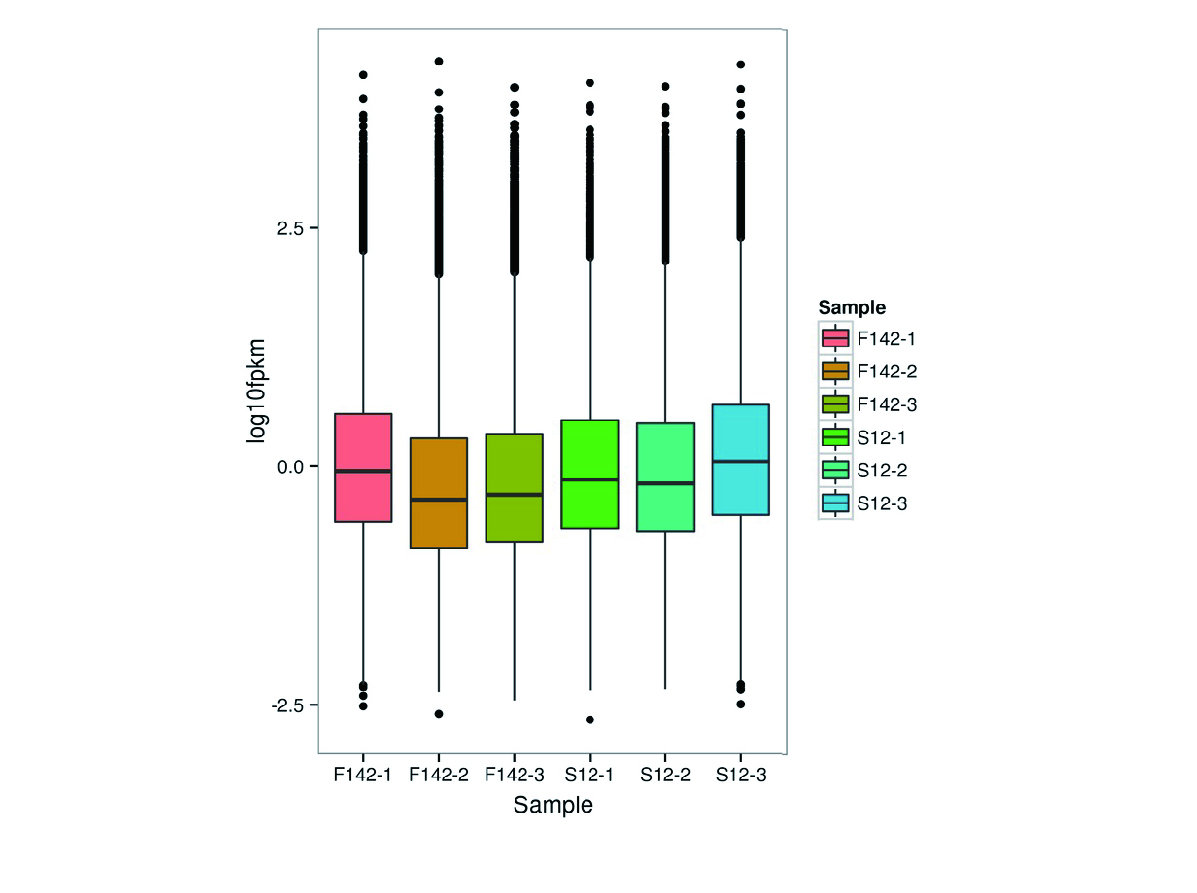


Figure S1. Statistical analysis of assembly reads mapped to the transcript.


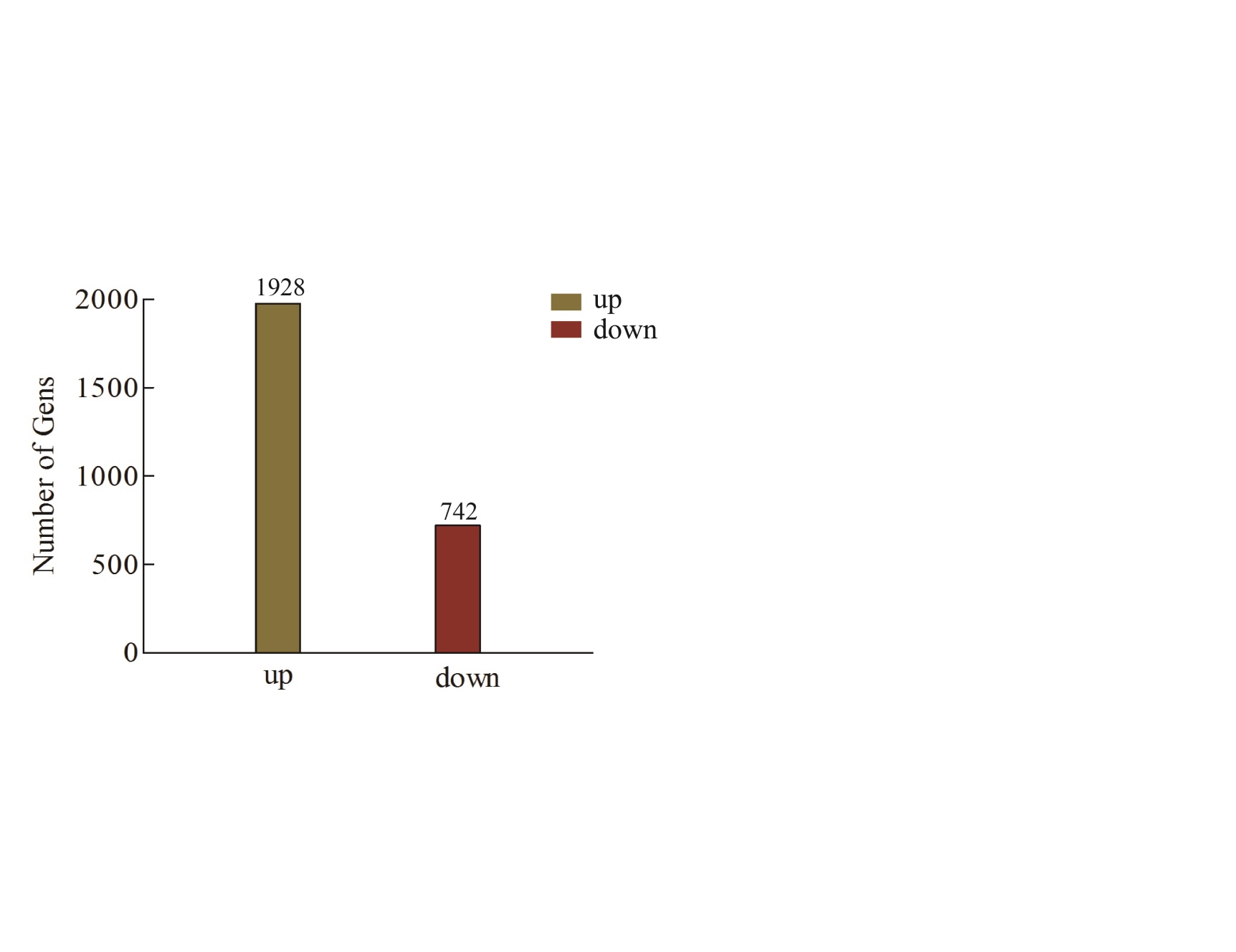


Figure S2. Statistical analysis of all identified differentially expressed genes (DEGs) in F142 and S12.


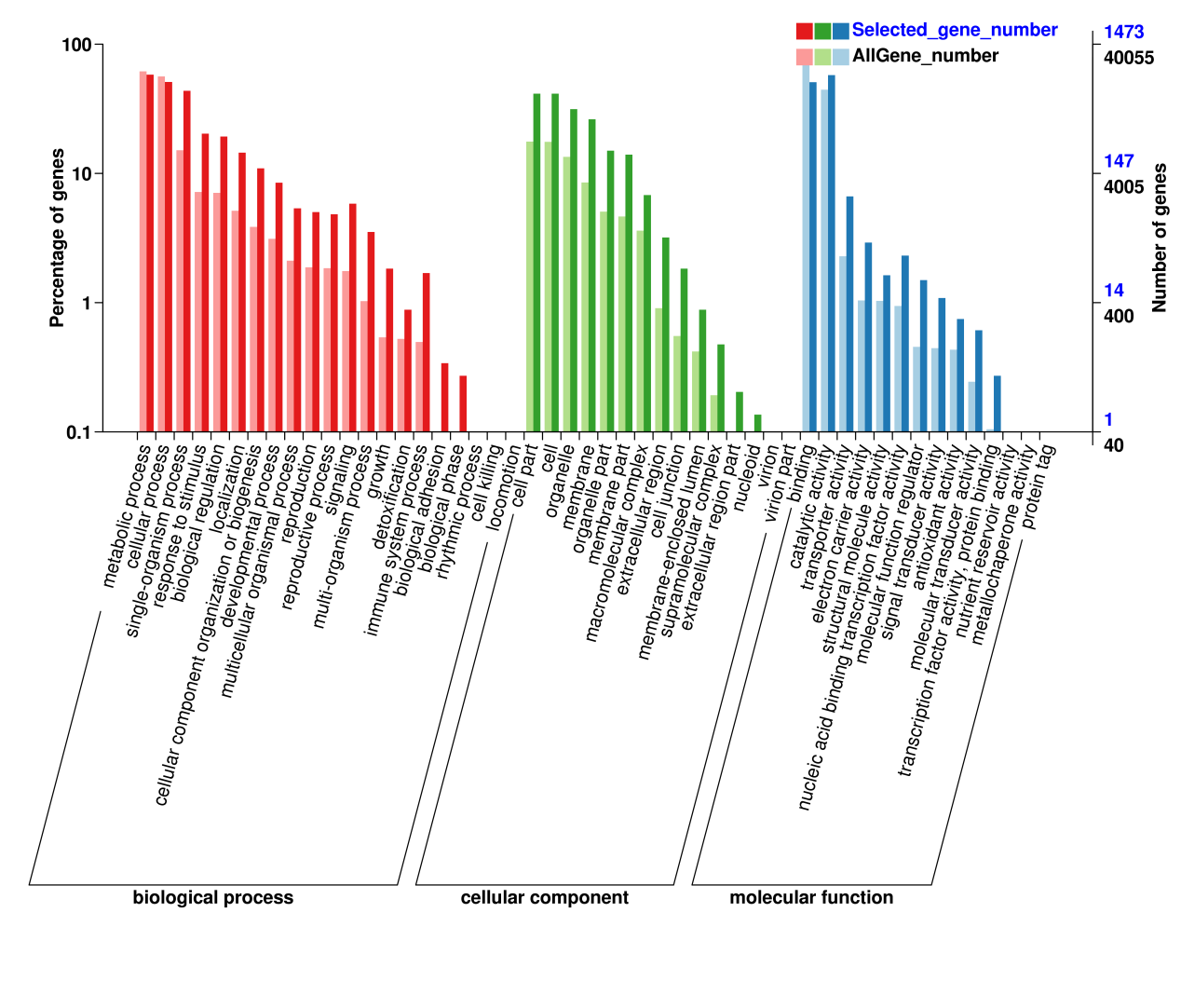
Figure S3. GO analysis of the total DEGs in molecular function, biological process, and cellular component in F142 vs. S12, respectively.


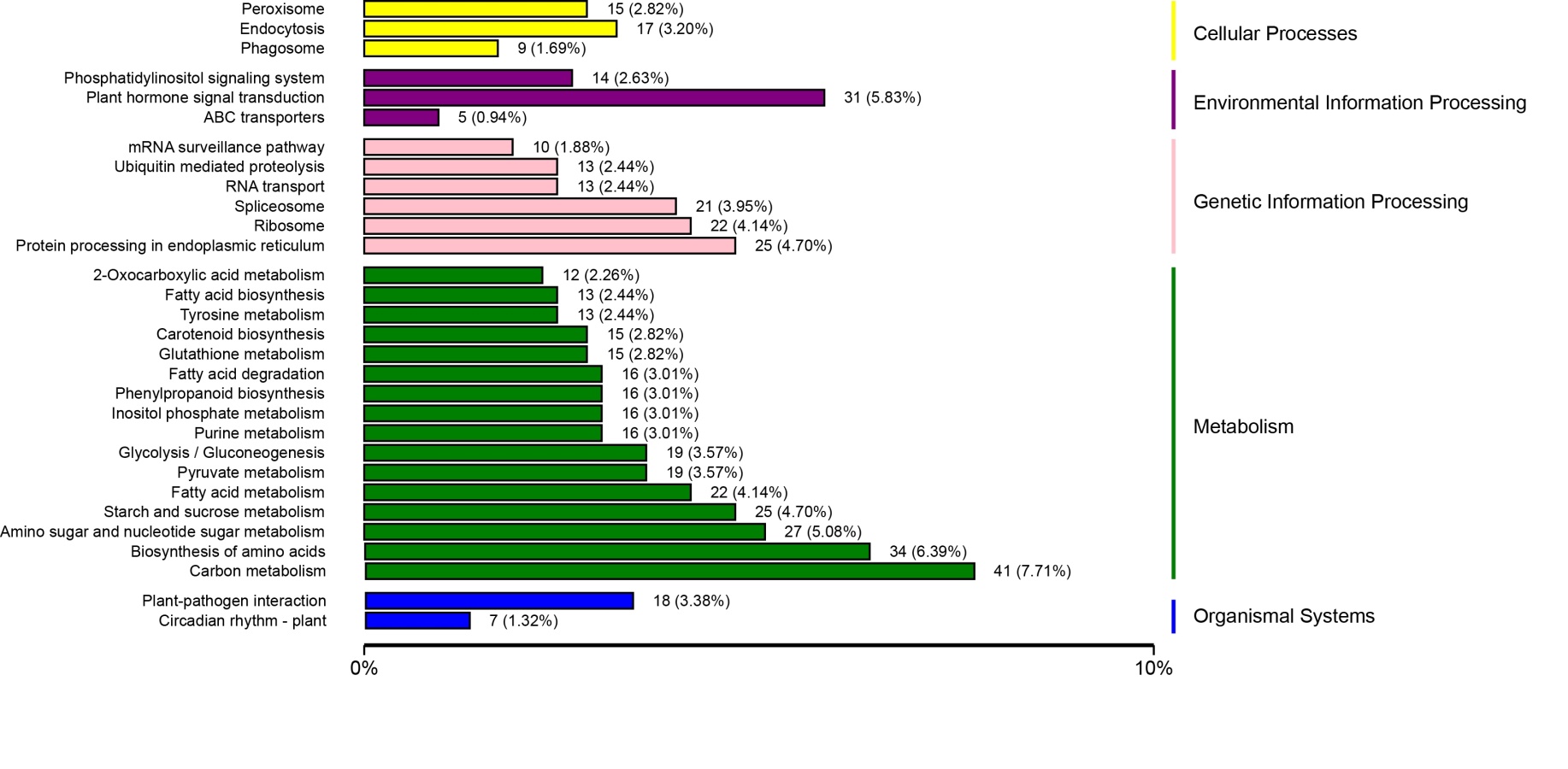


Figure S4. KEGG categories analysis of total DEGs in F142 vs. S12.


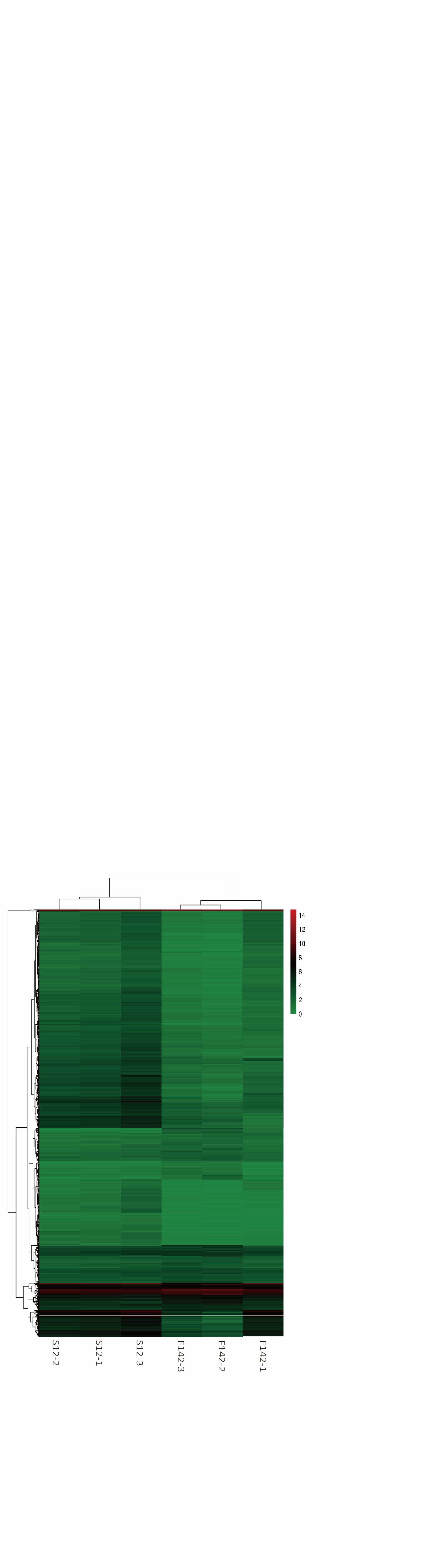


Figure S5. Expression analysis of total DEGs in F142 and S12.


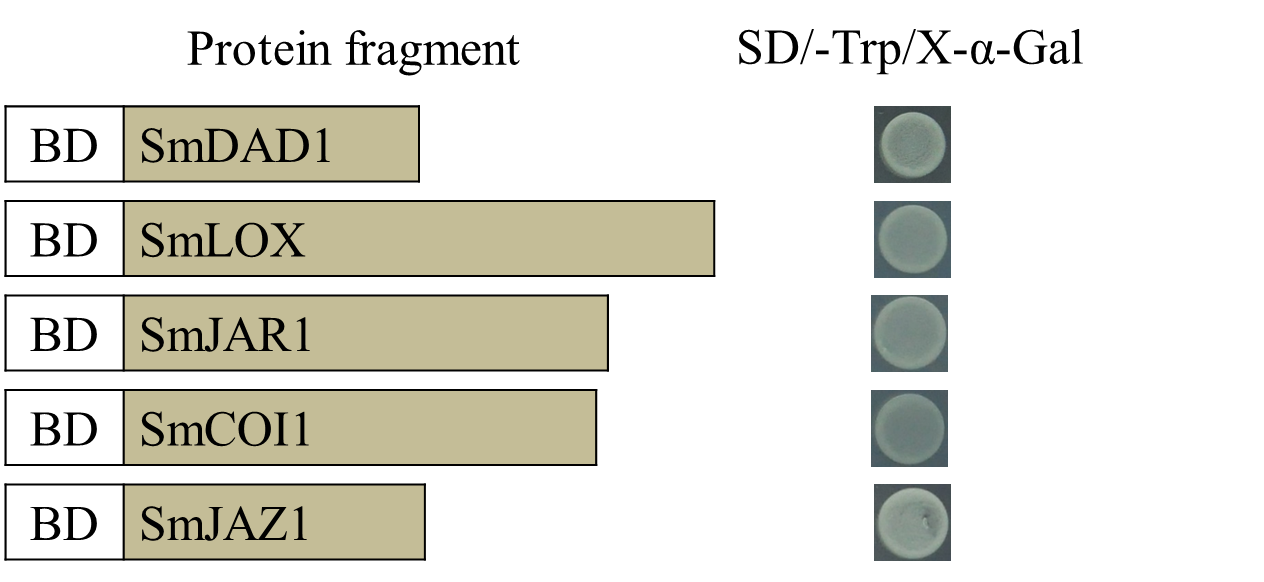


Figure S6. *Trans*-acting activity of recombinant plasmids in yeast cells based on the β-galactosidase activities
